# The biomechanical demands of standing yoga poses in seniors: The Yoga empowers seniors study (YESS)

**DOI:** 10.1186/1472-6882-13-8

**Published:** 2013-01-09

**Authors:** Man-Ying Wang, Sean S-Y Yu, Rami Hashish, Sachithra D Samarawickrame, Leslie Kazadi, Gail A Greendale, George Salem

**Affiliations:** 1 Division of Biokinesiology and Physical Therapy, University of Southern California, 1540 Alcazar St, Los Angeles, CA 90033, USA; 2 Division of Geriatrics, Geffen School of Medicine, University of California, Los Angeles, CA, USA

**Keywords:** Intervention, Lower-extremity, Biomechanics, Moment, EMG, Older adult

## Abstract

**Background:**

The number of older adults participating in yoga has increased dramatically in recent years; yet, the physical demands associated with yoga performance have not been reported. The primary aim of the Yoga Empowers Seniors Study (YESS) was to use biomechanical methods to quantify the physical demands associated with the performance of 7 commonly-practiced standing yoga poses in older adults.

**Methods:**

20 ambulatory older adults (70.7 + − 3.8 yrs) attended 2 weekly 60-minute Hatha yoga classes for 32 weeks. The lower-extremity net joint moments of force (JMOFs), were obtained during the performance of the following poses: Chair, Wall Plank, Tree, Warrior II, Side Stretch, Crescent, and One-Legged Balance. Repeated-measure ANOVA and Tukey’s post-hoc tests were used to identify differences in JMOFs among the poses. Electromyographic analysis was used to support the JMOF findings.

**Results:**

There was a significant main effect for pose, at the ankle, knee and hip, in the frontal and sagittal planes (p = 0.00 – 0.03). The Crescent, Chair, Warrior II, and One-legged Balance poses generated the greatest average support moments. Side Stretch generated the greatest average hip extensor and knee flexor JMOFs. Crescent placed the highest demands on the hip flexors and knee extensors. All of the poses produced ankle plantar-flexor JMOFs. In the frontal plane, the Tree generated the greatest average hip and knee abductor JMOFs; whereas Warrior II generated the greatest average hip and knee adductor JMOFs. Warrior II and One-legged Balance induced the largest average ankle evertor and invertor JMOFs, respectively. The electromyographic findings were consistent with the JMOF results.

**Conclusions:**

Musculoskeletal demand varied significantly across the different poses. These findings may be used to guide the design of evidence-based yoga interventions that address individual-specific training and rehabilitation goals in seniors.

**Clinical trial registration:**

This study is registered with NIH Clinicaltrials.gov #NCT 01411059

## Background

Yoga is an increasingly popular form of exercise activity for older adults, with senior participation in the US currently estimated at approximately 1 million practitioners [1]. Anecdotal and lay-journal reports affirm that regular yoga practice can increase strength, flexibility, balance, and physical capacity, improve emotional and spiritual wellness, and is relatively safe. Indeed, yoga has been recommended as a form of “*totalsolution*” exercise for seniors by the National Recreation and Park Association [2]. Despite these dramatic claims of improved function across a range of physiological and psychosocial domains, little is known about the physical demands, efficacy, and safety of yoga for older adults. Furthermore, compared to younger persons, older adults generally have lesser joint flexibility, strength and balance and a greater prevalence of osteoarthritis and back-pain syndromes (e.g. spinal-canal stenosis). Thus, seniors are at higher risk of developing musculoskeletal and neurological complications (e.g. strains, sprains, & impingements) when participating in yoga. An in-depth understanding of the demands placed on the musculoskeletal system by each of the yoga poses may reduce these undesirable side effects of yoga in seniors.

A primary aim of the YESS project was to quantify the physical demands associated with performance of the individual poses (asanas). And although an examination of individual yoga poses does not address the additional pantheon of attributes also associated with yoga practice, (e.g. breathing, meditation, chanting), ultimately this information can be used to design programs that are well-balanced, target a variety of functionally important muscle groups, and do not repeatedly overload the same musculoskeletal and articular tissues. Additionally, this information can be used to specifically target weak muscle groups and/or unload injured and healing structures. Like other exercise activities, the physical demands associated with yoga participation can be quantified by using biomechanical methods to estimate the net joint moments of force (JMOFs) and muscular activation patterns generated during performance of asanas [3]. While performing a pose, ground reaction forces acting on the body produce *external* JMOFs about the joints. These external JMOFs must be met by *internal* JMOFs, acting in the opposite direction and generated via muscular actions and ligamentous constraints, in order to maintain the position of the body’s center of mass and/or prevent collapse of the limbs. Internal JMOFs increase muscle loading and may stimulate beneficial adaptational responses (e.g. strength & endurance); however, JMOFs that are excessively high and/or acting in contraindicated directions, can result in the detrimental loading of articular, ligamentous & capsular structures— potentially exacerbating existing joint pathology (e.g. osteoarthritis; OA) [4,5].

The current report describes the lower-extremity physical demands (as measured by the JMOFs and electromyography [EMG]) associated with the performance of 7 standing yoga asanas that are commonly taught in senior yoga classes. The data are from the YESS study, which was a single-arm, non-masked, pre-post, intervention development study. A set of pre-specified, introductory poses were taught for 16 weeks, followed by 16 weeks of an intermediate pose series [6]. The results provided here come from the second series of poses, as these more closely approximated the “standard” (unmodified) forms of each asana. We hypothesized that the lower-extremity physical demands would vary across the lower-extremity joints, planes of motion, muscle groups, and individual limbs, among the standing poses.

## Methods

### Study design

YESS consisted of a 32-week yoga program with 2 phases: a 16-week beginning phase (*Series I*) and a 16-week intermediate phase (*Series II*). The study design and poses that were used in each phase have been detailed [6]. The primary biomechanical outcome variables were the net JMOFs during the performance of the individual yoga poses (asanas). Muscle activation patterns associated with the asanas, and adherence to the yoga program, were also assessed. Biomechanical data was collected at the Musculoskeletal Biomechanics Research Laboratory (MBRL) at the University of Southern California (USC). Subject recruitment and the yoga classes were conducted at the University of California Los Angeles (UCLA) and TruYoga studio (Santa Monica, CA), respectively. The USC and UCLA Institutional Review Boards approved the study protocol and all participants provided informed, written consent.

### Subjects

YESS was designed to design and test senior-adapted Hatha yoga poses intended to be suitable for ambulatory older adults. The study sample size was determined by power analysis (β = 0.8; p < 0.01) using the JMOF findings from a previous study [7]. Safety exclusions were adopted in order to decrease potential cardiovascular, musculoskeletal, and neurological risks to the participants; these included: active angina; uncontrolled hypertension (SBP > 160 or/and DBP > 90); high resting heart rate (greater than 90) or respiratory rate (greater than 24); unstable asthma or exacerbated chronic obstructive pulmonary disease; cervical spine instability or other significant neck injury; rheumatoid arthritis; unstable ankle, knee, hip, shoulder, elbow, or wrist joints; hemiparesis or paraparesis; movement disorders; peripheral neuropathy; stroke with residual deficit; severe vision or hearing problems; walker or wheelchair use; not able to attend in-person classes; has not had check-up by regular provider within 12 months (if not taking any prescription medications) or in the past 6 months (if any regular medicines taken). Participants also had to execute the following safety tests stably and independently: transition from standing to recumbent on the floor and reverse; lift both arms to shoulder level; stand with feet side-by-side for 30 seconds; and stand with feet hip-width apart for 60 seconds.

### Yoga program

Participants attended 2 60-minute yoga sessions per week, for 32 weeks. The yoga program was developed by the research team, which included an experienced yoga therapist (RYT-500), a geriatrician, an exercise physiologist/biomechanist, and a physical therapist. In general, the program was an adapted form of Hatha yoga [8]. Two sets of poses (*Series I* and *Series II*) were taught. We report herein the biomechanical findings assessed after the completion of the second series, because the second series was more homogenously performed than that was the first series. This increasing homogeneity in pose form over time is inherent in working with senior participants, who initially exhibit a broad range of yoga-performance capabilities, related to each subject’s strength, flexibility, balance, overall fitness and group-exercise experience. This heterogeneity in capability necessitates greater pose modification to avoid harm. However, the second series poses build on the training achieved in the first series. Thus, they require fewer modifications from the standard forms of the asanas. By the end of the second series training period (32-weeks), all participants could perform all second series poses. Thus, the present analysis examines 7 standing poses, performed at the completion of the series two training period. These are: Chair, Wall Plank, Tree, Warrior II, Side Stretch, Crescent, and One-Legged Balance. A detailed description of the poses, including photographs, can be found in the report by Greendale et al. [6].

### Kinematics and kinetics

Reflective markers were placed on a head band and over the following anatomical landmarks of the lower- and upper-extremities bilaterally: first and fifth metatarsal heads, malleoli, femoral epicondyles, greater trochanters, acromions, greater tubercles, humeral epicondyles, radial and ulnar styloid processes, and third metacarpal heads. Markers were also attached to the spinous process of the 7th cervical vertebrae (C7), jugular notch, L5/S1, bilateral iliac crests, and bilateral posterior superior iliac spines, in order to define the trunk and pelvis. Based on these markers, a total of 15 body segments were modeled: the head, trunk, pelvis, the upper arms, forearms, hands, thighs, shanks, and feet. Non-collinear tracking marker plates were placed on each of these segments to track segmental position during the poses, using previously documented procedures [9,10].

**Figure 1:**
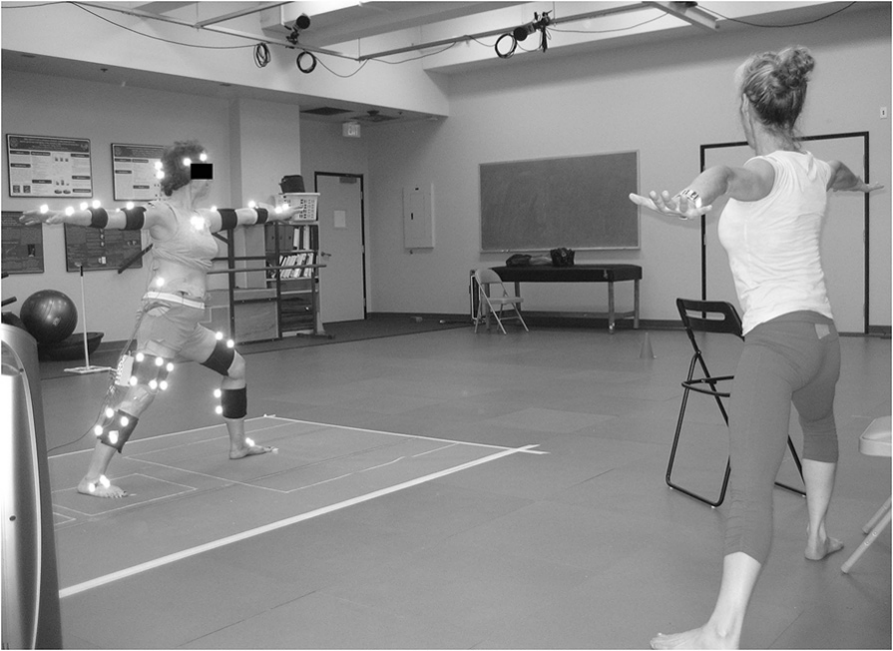
An instrumented participant performing the Warrior II pose while guided by the yoga instructor.

**Figure 2:**
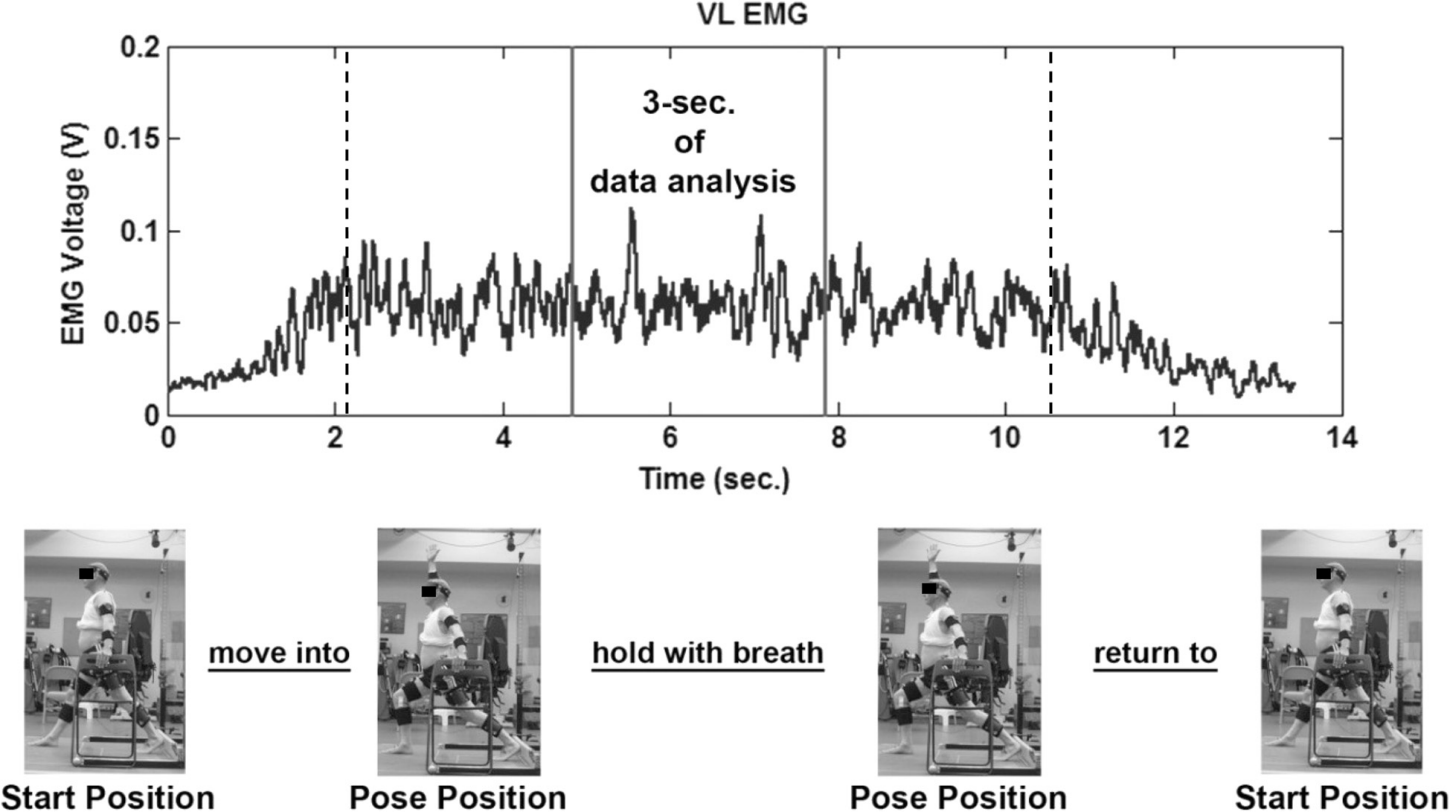
**EMG signal of the Vastus Lateralis during performance of the Crescent pose. **Data analysis was conducted on the middle 3 seconds (between solid lines), during the static portion of the pose (between dashed lines).

Once instrumented, the subjects performed their poses while guided by the yoga instructor (Figure 1^a^). Props, including foam blocks (One-legged Balance) and a chair (Side Stretch and Crescent) were used in the same manner as they were used during the participant’s regular practice sessions in the yoga studio. A firm but portable clear plexiglas wall, which permitted capture of the markers, was positioned for the Wall Plank pose. For the single-limb poses, measurements were taken on the dominant limb. For poses requiring bilateral limb support, each foot was positioned on an independent force platform. For each pose, the participant was instructed to begin in a starting position, move smoothly into the pose, hold the pose while taking a full breath, then return back to the original position. The instructor performed each pose along with the participant in order to provide visual cueing. Two trials of each pose were collected and averaged; data during the middle 3 seconds of each pose was used for analysis (Figure 2^a^). Data during the middle 3 seconds, while holding the pose, was used for analysis. For poses that involved asymmetric positioning of the 2 support limbs (e.g. Side Stretch, Crescent, and Warrior II asanas), measurements were obtained by repeating the poses, initially with the dominant limb in the lead (front) position and subsequently in the trailing (back) position. Because the JMOFs varied considerably between the leading and trailing limbs, the limbs were considered separately. Thus, Side Stretch, Crescent, and Warrior II asanas were subdivided into Leading- and Trailing-limb poses (e.g. Crescent Leading and Crescent Trailing). The subjects also completed 2 successful walking trials at their self-selected “comfortable speed”. The walking trials provided a well-studied, stereotypical activity for comparisons of the JMOFs and muscle activation patterns with the respective poses.

Three-dimensional coordinates of the body segments were recorded by an 11-camera system at 60 Hz (Qualisys, Gothenburg, Sweden). Ground reaction forces were measured from separate force platforms at 1560 Hz (AMTI, Watertown, MA). Data processing software (Visual 3D, C-Motion, Inc. Germantown, MD) was used to process the raw coordinate data and compute the segmental kinematics. The principle moments of inertia were determined from the subject’s total body weight, segment geometry, and anthropometric data. Using standard inverse dynamics techniques consistent with the International Society of Biomechanics recommended coordinate systems, the JMOFs in the sagittal and frontal planes, for the ankle, knee and hip, were calculated from the inertial properties, segmental kinematics, and ground reaction forces [11,12]. JMOFs were normalized to each subject’s bodyweight in kilograms (kg). Additionally, a *support moment*, calculated as the sum of the ankle, knee, and hip sagittal plane JMOFs, was determined for each pose [13,14]. These instrumentation and data-processing techniques have previously been used in our laboratory to assess exercise activities in older adults with high reliability (Cronbach’s alpha = 0.98) [15].

### Electromyography (EMG)

Surface EMG signals of the gluteus medius (GMED), hamstrings (HAMS), vastus lateralis (VL), and gastrocnemius (GAS) muscles were collected using active surface electrodes (Motion Lab Systems, Baton Rouge, LA). Data from the dominant limb were recorded at 1560 Hz. Standard procedures for older-adult participants including preparation of the skin and electrode placement were employed [16]. The EMG signals were filtered according to ISEK standards, including, notch filtering at 60 Hz, and band-pass filtering between 20 and 500 Hz. A root mean square smoothing algorithm, with a 75-millisecond constant window, was used to smooth the EMG data [17]. EMG smoothing, processing and normalization were performed using a custom written MATLAB program (MathWorks, Natick, MA).

### Data analysis

Visual inspection was used to select the top 4–5 ranked poses for statistical comparison, across each joint, plane of motion, and direction. Parametric distribution of the JMOFs was confirmed by analyzing the skewness and kurtosis of the data. Repeated-measure ANOVA omnibus tests were used to identify significant differences in the JMOFs within each cluster of the top 4–5 ranked poses. When the results were significant, Tukey’s post-hoc tests were used to examine the pairwise comparisons. Additionally, Cohen’s d effect sizes (small *d* = 0.2; medium *d* = 0.5; large *d* = 0.8) are reported for all statistically significant post-hoc comparisons [18]. Statistical analysis was conducted via PASW Statistics 18 (IBM SPSS Statistics, Armonk, NY) and significance level was set at α = 0.05. The EMG data was used to support the primary JMOF findings; formal statistical analyses were not conducted on the EMG data.

## Results

### Subjects

Twenty participants (6 men and 14 women) completed the 32-week program and attended the biomechanical data collection of the Series II poses. Their average age, height, weight, and body mass index was 70.7 ± 3.8 years, 1.67 ± 0.07 m, 71.3 ± 14.6 kg, and 25.3 ± 4.1 kg/m^2^, respectively. On average, the participants attended 85.4% ± 7.6% and 80.3% ± 13.2% of the Series I and Series II classes, respectively.

### Support moment

**Figure 3:**
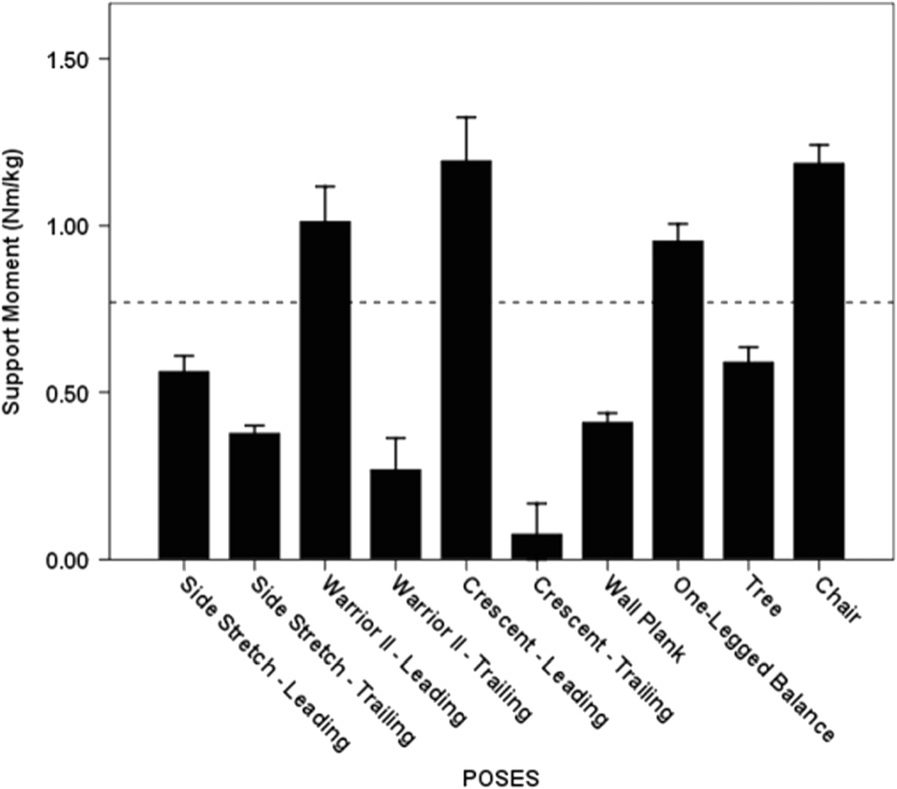
**Average Support moments. **The dashed line indicates the average peak support moment generated during walking at a self-selected speed. The whiskers represent standard errors. There was no statistically significant difference among the 4 poses generating the greatest average support moments (p = 0.07 – 1.00).

The Crescent Leading, Chair, Warrior II Leading, and One-legged Balance poses generated the greatest average support moments; however, significant differences were not evident among these poses (p = 0.07 – 1.00; Figure 3). The average support moment generated across these 4 poses (1.09 ± 0.40 Nm/kg) was 183% greater than the average support moment generated across the remaining 6 poses and 42% greater than the peak support moment generated during self-selected walking (0.77 ± 0.36 Nm/kg; p < 0.001).

### Net joint moments of force (JMOF)

**Figure 4:**
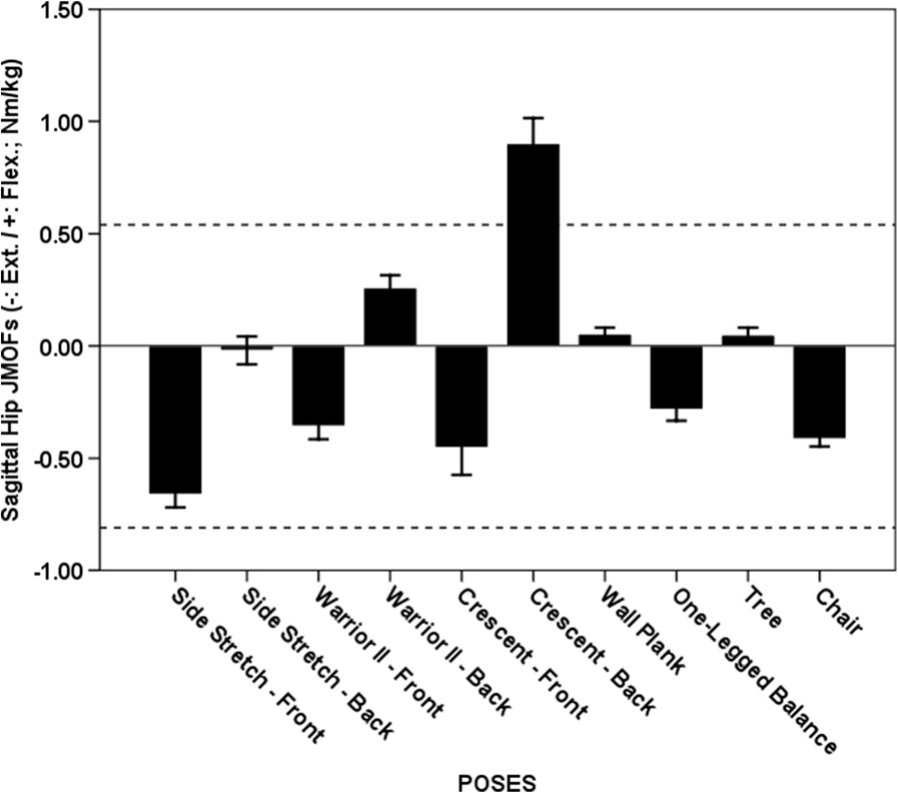
**Average Hip JMOFs in the sagittal plane. **The dashed lines indicate the average peak JMOF generated during walking at a self-selective speed. The whiskers represent standard errors. Average flexor JMOFs: Crescent Trailing > Warrior II Trailing, Wall Plank & Tree (p < 0.001, *d* = 1.7 – 2.4); Warrior II Trailing > Tree (p = 0.043, *d* = 1.0). Average extensor JMOFs: Side Stretch Leading > Chair (p = 0.018, *d* = 1.2), Warrior II Leading (p = 0.002, *d* = 1.2) & One-legged Balance (p < 0.001, *d* = 1.5).

**Figure 5:**
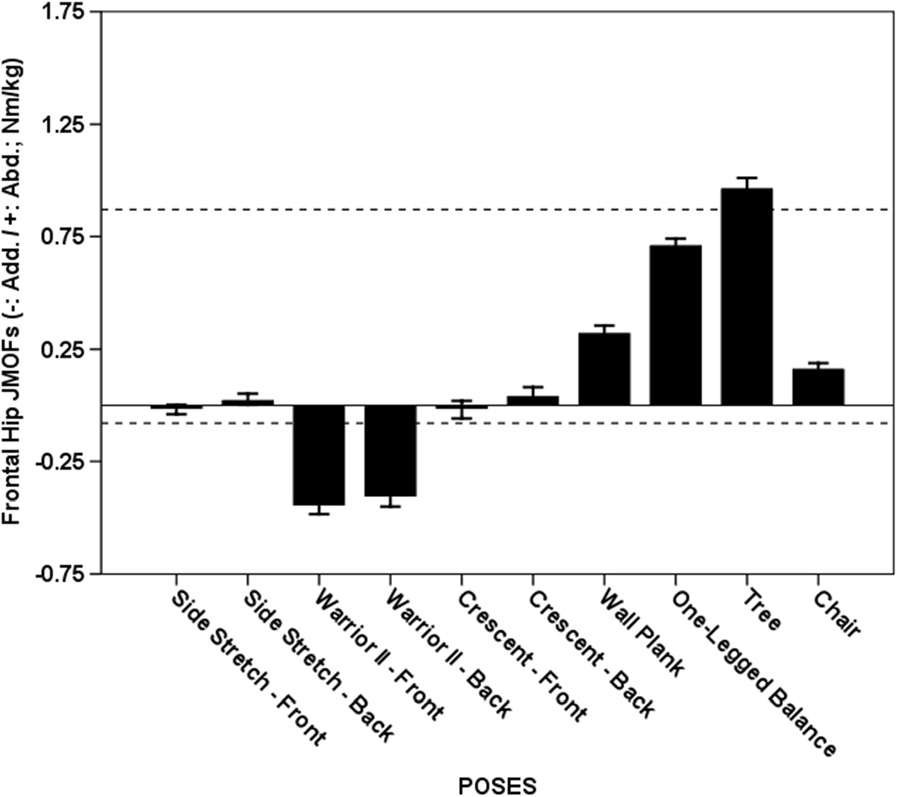
**Average Hip JMOFs in the frontal plane. **The dashed lines indicate the average peak JMOF generated during walking at a self-selective speed. The whiskers represent standard errors. Average abductor JMOFs: Tree > One-legged Balance, Wall Plank & Chair (p < 0.001, *d* = 1.6 – 5.3); One-legged Balance > Wall Plank & Chair (p < 0.001, *d* = 3.0 – 4.9); Wall Plank > Chair (p < 0.001, *d* = 1.4). Average adductor JMOFs: Warrior II Leading > Crescent Leading & Side Stretch Leading (p < 0.001, *d* = 2.4 – 2.6); Warrior II Trailing > Crescent Leading & Side Stretch Leading (p < 0.001, *d* = 2.1 – 2.2).

**Figure 6:**
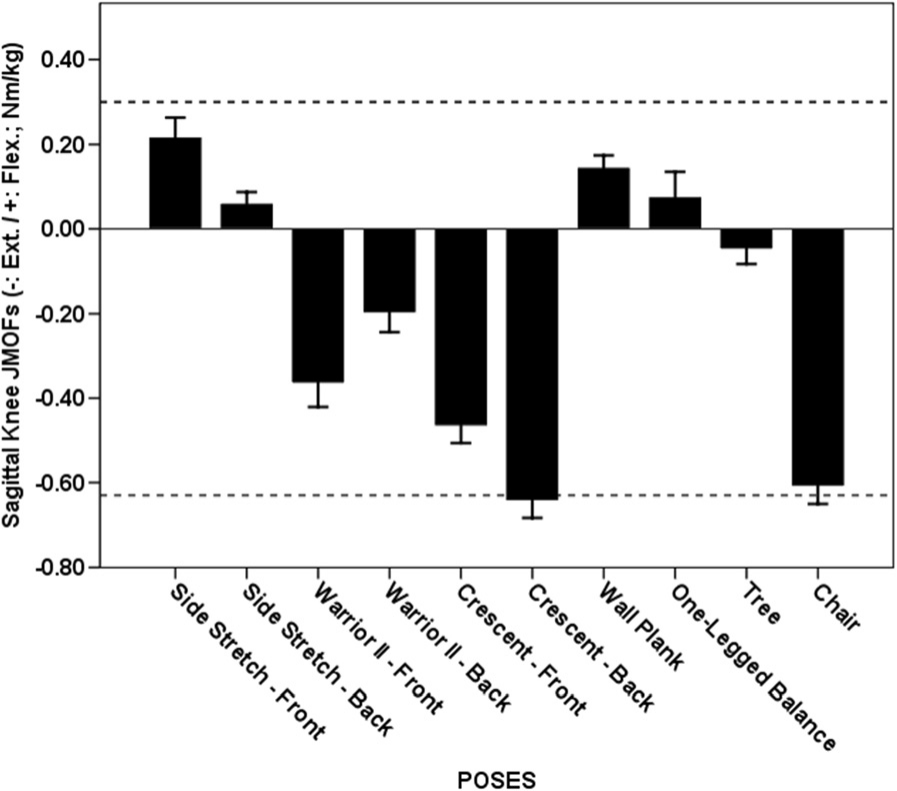
**Average Knee JMOFs in the sagittal plane. **The dashed lines indicate the average peak JMOFs generated during walking at a self-selective speed. The whiskers represent standard errors. Average flexor JMOFs: Side Stretch Leading > One-legged Balance (p = 0.048, *d* = 0.6) & Side Stretch Trailing (p = 0.023, *d* = 1.0). Average extensor JMOFs: Crescent Trailing > Crescent Leading (p = 0.006, *d* = 0.9) & Warrior II Leading (p < 0.001, *d* = 1.2); Chair > Crescent Leading (p = 0.035, *d* = 0.8)& Warrior II Leading (p < 0.001, *d* = 1.1).

**Figure 7:**
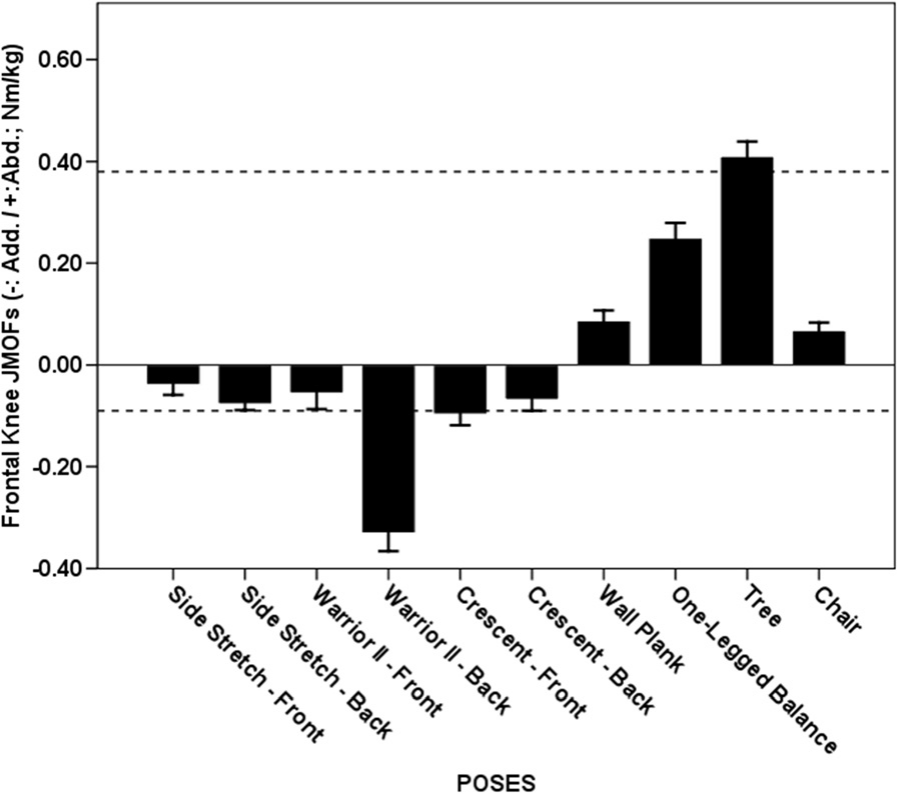
**Average Knee JMOFs in the frontal plane. **The dashed lines indicate the average peak JMOFs generated during walking at a self-selective speed. The whiskers represent standard errors. Average abductor JMOFs: Tree > One-legged Balance, Wall Plank & Chair (p < 0.001, *d* = 1.2 – 3.3); One-legged Balance > Wall Plank & Chair (p < 0.001, *d* = 1.4 – 1.7). Average adductor JMOFs: Warrior II Trailing > Crescent Leading, Side Stretch Trailing, and Crescent Trailing (p < 0.001, *d* = 1.7 – 2.1).

**Figure 8:**
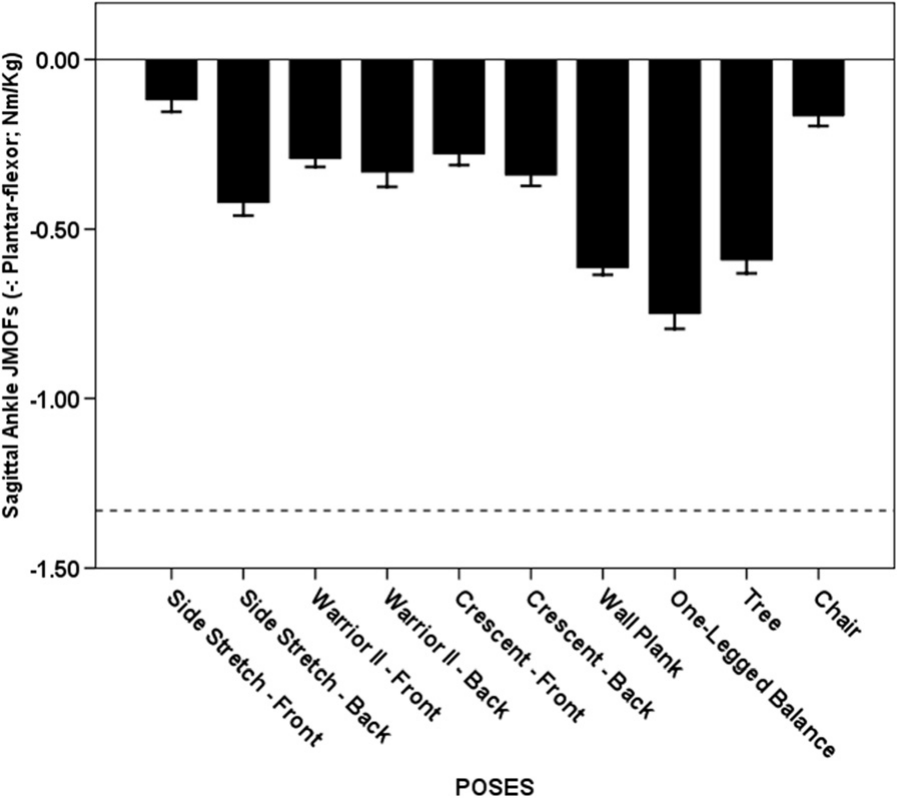
**Average Ankle JMOFs in the sagittal plane. **The dashed line indicates the average peak ankle JMOFs generated during walking at a self-selected speed. The whiskers represent standard errors. Average plantar-flexor JMOFs: One-legged Balance > Wall Plank (p = 0.015, *d* = 0.9), Tree (p = 0.004, *d* = 0.9), & Side Stretch Trailing (p < 0.001, *d* = 1.9); Wall Plank > Side Stretch Trailing (p < 0.001, *d* = 1.6); Tree > Side Stretch Trailing (p = 0.002, *d* = 1.1).

**Figure 9:**
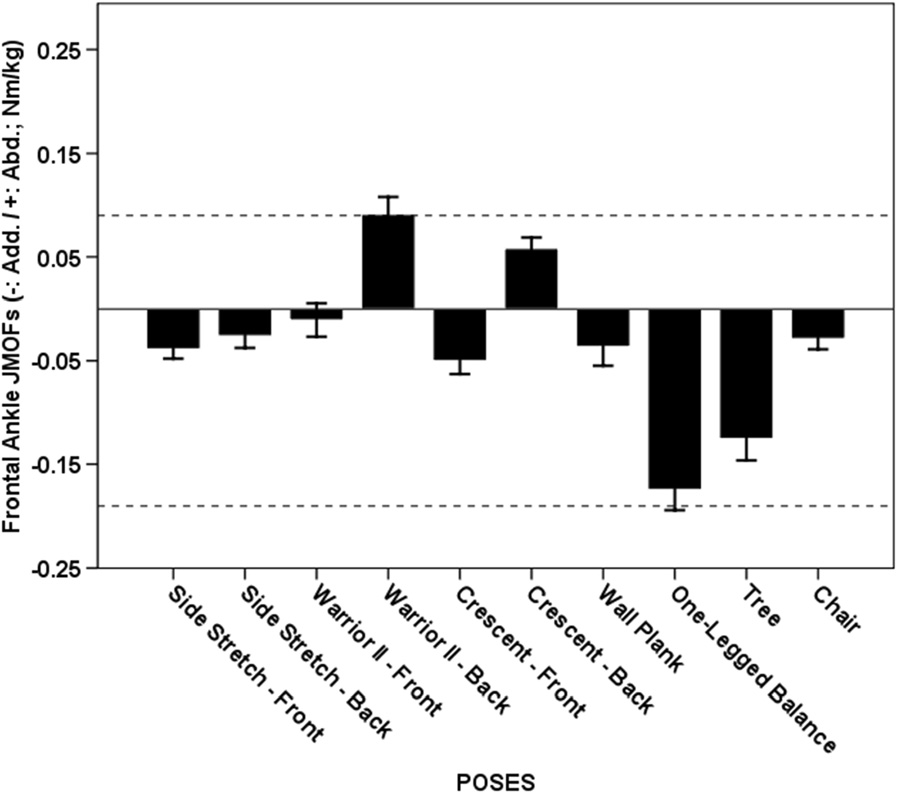
**Average Ankle JMOFs in the frontal plane. **The dashed line indicates the average peak ankle JMOFs generated during walking at a self-selected speed. The whiskers represent standard errors. Average abductor JMOFs: Warrior II Trailing > Crescent Trailing (p = 0.032, *d* = 0.4). Average adductor JMOFs: One-legged Balance > Tree (p = 0.039, *d* = 0.4), Crescent Leading (p < 0.001, *d* = 1.6) & Side Stretch Leading (p < 0.001, *d* = 1.9); Tree > Crescent Leading & Side Stretch Leading (p < 0.001, *d* = 1.0 – 1.3).

Results from the repeated-measure ANOVA omnibus tests indicated a significant main effect of pose within each cluster of top ranked 4–5 poses for all joints, planes of motion, and directions (p = 0.00 – 0.03). Significant differences between poses were identified by the post-hoc tests and are illustrated in Figures 4, 5, 6, 7, 8, 9. The peak joint moments generated during the stance phase of the self-selected walking trials are also illustrated in these figures.

### Hip extensor JMOF

Five poses – Side Stretch Leading, Warrior II Leading, Crescent Leading, One-legged Balance and Chair– generated average hip extensor JMOFs that were 21-82% of the peak hip extensor JMOFs generated during self-selected walking (Figure 4). Post-hoc analysis revealed that the average Side Stretch Leading hip extensor JMOF was significantly greater than the Chair (61%; p = 0.018, *d* = 1.2), Warrior II Leading (86%; p = 0.002, *d* = 1.2), and One-legged Balance (134%; p < 0.001, *d* = 1.5) JMOFs. No other significant hip extensor JMOF differences were identified. In general, the EMG findings were in agreement with the JMOF findings—all poses that generated an appreciable hip extensor JMOF also generated appreciable HAMS EMG activity. The HAMS average EMG activity of the top 5 poses were 18-86% of the peak HAMS EMG activity generated during self-selected walking.

### Hip flexor JMOF

Only 4 poses generated a hip flexor JMOF—Warrior II Trailing, Crescent Trailing, Wall Plank, and Tree (Figure 4). Post hoc analysis revealed that the hip flexor JMOF during Crescent Trailing was 250–1,700% greater than the other 3 poses (p < 0.001, *d* = 1.7 – 2.4). It was also 69% greater than the peak JMOF produced during self-selected walking. EMG data was not collected on the hip flexors.

### Hip abductor JMOF

The 4 poses that generated the greatest average hip abductor JMOFs were the Tree, One-legged Balance, Wall Plank, and Chair (Figure 5). Post hoc analysis revealed that all 4 poses were significantly different from each other (p < 0.001) and the between-pose Cohen’s *d* ranged from 1.4 to 5.3. Compared with self-selected walking, the Tree pose generated a 12% greater hip abductor JMOF, whereas the One-legged Balance, Wall Plank, and Chair poses generated JMOFs that were 18 - 81% less than the average peak JMOF generated during self-selected walking. Consistent with the JMOF findings, the GMED, a primary hip abductor, was active during performance of all the poses that generated hip abductor JMOFs. These GMED activity levels, however, were only 12 - 38% of those recorded during self-selected walking.

### Hip adductor JMOF

The Warrior Leading and Trailing poses were the only poses that generated appreciable hip adductor JMOFs (Figure 5). These JMOFs were approximately 4 times greater than the average peak JMOF produced during self-selected walking. EMG data was not collected on the hip adductor muscles.

### Knee extensor JMOF

The Crescent Trailing and Chair poses generated the greatest average knee extensor JMOFs, which were significantly greater (31-76%, *d* = 0.8 – 1.2) than the 3^rd^- and 4^th^-ranked poses—Crescent Leading and Warrior II Leading (Figure 6). The knee extensor JMOFs engendered by Crescent Trailing and Chair were similar to the average peak JMOF generated during self-selected walking; however, the average activation level of the VL, a primary knee extensor, during the yoga poses was only 33-49% of the peak activity generated during the walking trials.

### Knee flexor JMOF

Four poses - Side Stretch Leading, Side Stretch Trailing, Wall Plank, and One-legged Balance - generated knee flexor JMOFs (Figure 6), which were 20.0% - 73.3% of the average peak JMOF generated during self-selected walking. Of these 4 poses, the Side Stretch Leading JMOF was approximately 2 and 2.5times greater than the One-legged Balance (p = 0.048, *d* = 0.6) and Side Stretch Trailing (p = 0.023, *d* = 1.0) JMOFs, respectively. Consistent with the JMOF results, all 4 poses generated appreciable HAMS muscle activity. The average HAMS EMG activity during these 4 poses was 14-86% of the peak activity generated during self-selected walking. The EMG activity of the GAS, also a knee flexor, ranged from 5-44% of the peak GAS activity produced during self-selected walking.

### Knee abductor JMOF

Four poses engendered knee abductor JMOFs—Tree, One-legged Balance, Wall Plank, and Chair (Figure 7). The Tree pose generated the largest JMOF, which was 65- 503% greater than the other 3 poses (p < 0.001, *d* = 1.2 – 3.3). It was also 8% greater than the peak JMOF generated during self-selected walking. The knee abductor JMOFs of the 3 other poses were only 18-66% of the peak JMOF generated during self-selected walking.

### Knee adductor JMOF

Consistent with the *hip* adductor findings, Warrior II Trailing pose also produced the highest knee adductor JMOF (Figure 7), which was 240-385% greater than the JMOFs produced by the other poses (p < 0.001, *d* = 1.7 – 2.1). The knee adductor JMOF of the Warrior II Trailing was also 267% greater than the peak JMOF produced during self-selected walking.

### Ankle plantar-flexor JMOF

All of the analyzed poses produced ankle plantar-flexor JMOFs (Figure 8). The plantar-flexor JMOF of the One-legged Balance pose was 22-77% greater than the other 3 highest-ranking poses (Wall Plank, Tree, and Side Stretch Trailing) (p < 0.001 – p = 0.015, *d* = 0.9 – 1.9). The top 4 ranked poses generated plantar-flexor JMOFs that were only 32-56% of the peak plantar-flexor JMOF generated during self-selected walking. The GAS, a primary ankle plantar-flexor, was active during all the poses. Consistent with the JMOF results, GAS EMG activity during the top 4 ranked poses was only 5-44% of the peak activity generated during self-selected walking.

### Ankle abductor (evertor) JMOF

Only 2 poses, Warrior II Trailing and Crescent Trailing poses, produced an ankle abductor JMOFs (Figure 9) and these JMOFs were 100% and 67% of the peak abductor JMOF generated during self-selected walking, respectively. The Warrior II Trailing pose produced 57% greater abductor JMOF than the Crescent Trailing pose (p = 0.032, *d* = 0.4).

### Ankle adductor (invertor) JMOF

The One-legged Balance and Tree poses generated the greatest ankle adductor JMOFs, which were 89% and 68% of the peak JMOF generated during self-selected walking, respectively (Figure 9). The ankle adductor JMOF of the One-legged Balance pose was 39% greater than the Tree pose (p = 0.039, d = 0.4). These 2 poses produced 150-1500% greater ankle adductor JMOFs than all other poses did (P < 0.001, *d* = 1.0 – 2.0). Supporting the JMOF findings, GAS (an adductor agonist) EMG activity was greatest during the One-legged Balance and Tree poses; however, this activity was only 44% and 36% respectively of the peak GAS activity generated during self-selected walking (Table 1).

**Table 1:** Average EMG activity (%)*

**Pose/muscle**	**GAS**	**HAMS**	**VL**	**GMED**
**Crescent Trailing**	5.6 ± 3.2	13.9 ± 11.9	32.7 ± 25.2	11.0 ± 6.3
**Crescent Leading**	8.6 ± 5.6	19.4 ± 15.9	43.2 ± 38.4	15.7 ± 9.2
**Chair**	4.6 ± 2.8	19.2 ± 16.0	49.2 ± 44.7	13.5 ± 10.2
**Wall Plank**	10.0 ± 6.8	13.9 ± 16.9	14.7 ± 28.4	12.1 ± 17.8
**Side Stretch Trailing**	4.5 ± 2.6	12.8 ± 9.5	23.3 ± 28.5	11.4 ± 6.3
**Side Stretch Leading**	15.1 ± 12.6	22.8 ± 21.4	11.7 ± 8.3	12.1 ± 12.8
**One-leg Balance**	43.9 ± 22.6	85.9 ± 112.0	36.2 ± 35.6	37.9 ± 25.2
**Tree**	35.7 ± 15.3	36.3 ± 51.3	38.6 ± 39.0	24.6 ± 17.4
**Warrior II Trailing**	10.0 ± 4.3	17.1 ± 16.3	31.9 ± 24.5	9.4 ± 5.3
**Warrior II Leading**	8.9 ± 6.3	18.3 ± 14.4	43.8 ± 37.5	16.4 ± 12.1

*Data are expressed as a percentage of the peak EMG activity generated during self-selected walking. *GAS *= gastrocnemius, *HAMS *= hamstrings, *VL *= vastus lateralis, *GMED *= gluteus medius.

## Discussion

This study newly characterizes the physical demands of 7 standing yoga poses in a sample of older adults who had been trained for 32 weeks. We quantified the JMOFs associated with the performance of these yoga poses (10 including Leading and Trailing limbs). A significant main effect for pose was found across all of the JMOFs examined, suggesting significantly different musculoskeletal demands among the top 4–5 ranked poses.

### Support moment

The Crescent Leading, Chair, Warrior II Leading and One-legged Balance poses generated the greatest support moments and these were appreciably greater than the peak support moment associated with self-selected walking. Poses which generate a relatively high support moment may be thought of as good “comprehensive” asanas because they simultaneously target 3 functionally important muscle groups which prevent collapse of the center of mass during standing and walking—the hip extensors, knee extensors, and ankle plantar-flexors [13]. Moderate to high extensor JMOFs at the hip and knee were the primary contributors to the large support moments associated with Crescent Leading, Chair, and Warrior II Leading poses. All 3 of these poses involved a flexed knee position and the body center of mass was located relatively far from the hip and knee joint centers. The large support JMOF observed during the One-legged Balance pose, a free one-legged standing pose with the non-weight bearing limb flexed at the hip, was primarily due to the high ankle plantar-flexor JMOF. In addition to targeting the hip and knee extensors and ankle plantar-flexors, this pose may offer additional balance-training advantages over the other 3 poses because it requires a reduced base of support (one limb only). Interventions that incorporate single-limb standing activities increase balance capabilities [19], and decrease falls and fall risk [20].

### Sagittal plane JMOFs

The Side Stretch Leading pose generated the greatest hip extensor and knee flexor JMOFs. Consequently, this pose would be a good selection for targeting the hamstring muscles—which both extend the hip and flex the knee during concentric actions. Among all the poses examined, the highest level of EMG activation of the hamstrings was observed during Side Stretch Leading. The Crescent Trailing pose was associated with the largest hip flexor and knee extensor JMOFs. Thus, this pose would be a good selection for targeting the quadriceps muscles and hip flexors (iliacus, psoas major, and the hip adductors). Moreover, this asana would be an ideal pose for training of the rectus femoris muscle, a biarticular knee extensor *and* hip flexor. All of the poses analyzed produced ankle plantar-flexor JMOFs with the highest JMOF observed during One-legged Balance. These findings have important clinical implications and suggest that additional activities and/or poses would have to be included in order to target the ankle dorsiflexors. The ankle dorsiflexors “lift” the foot (i.e. dorsiflex the ankle) during the swing-phase of gait in order to permit sufficient clearance of the toes. Seniors with insufficient dorsiflexor strength or muscular endurance are thus at risk of tripping [21,22].

### Frontal plane JMOFs

The Tree pose generated the greatest hip and knee abductor JMOFs; whereas, Warrior II generated the greatest hip and knee adductor JMOFs. The frontal JMOFs during Warrior II were also greater than the peak JMOFs generated during self-selected walking. Several studies have quantified the relations among hip abductor performance, osteoarthritis progression [23], balance, and fall risk [24-26]. Thus, the Tree, and to a lesser extent the One-Legged Balance pose, would appear to be good selections for improving balance and reducing fall risk. Gluteus medius EMG findings also support that these poses target the hip abductors. To our knowledge, associations among hip *adductor* performance, balance capabilities, and fall risk, have not been examined.

Our findings regarding the frontal-plane JMOFs about the knee may have exceptionally important implications for instructors and clinicians designing programs for individuals with knee pain and/or pathology. Load demands in the frontal plane of the knee joint are primarily supported by passive structures (i.e. ligaments and the joint capsule), and not by muscle. Moreover, high frontal-plane JMOFs at the knee are associated with high compressional forces on the opposite side of the joint [4,27] and these high compressional forces, in turn, can exacerbate existing OA, accelerate articular cartilage degeneration, and increase pain [5,23,28,29]. While Tree and Warrior II poses were the best candidates for improving hip abductor and adductor performance, respectively, they also generated the greatest torque about the medial and lateral knee joint. For example, the average knee adductor JMOF produced by Warrior II was 267% greater than the *peak* knee JMOF produced during self-selected walking—suggesting that for long-term yoga practice, this pose may need to be modified or substituted for seniors with knee pain or pathology.

Across all poses, the JMOFs at the ankle were smaller than the average peak JMOF produced during self-selected walking. Only Crescent Trailing and Warrior II Trailing poses generated ankle abductor JMOFs. Large ankle adductor JMOFs were observed with One-legged Balance and Tree. Both ankle invertor and evertor strength are important for balance and safe ambulation, and they are related to performance in the timed up-and go test and Berg Balance Scale [30]. Practicing the aforementioned poses will likely target the ankle invertors and evertors and potentially improve balance associated with various daily living activities.

When *exercise* is prescribed, biomechanical assessment may be used to quantify the magnitude of the musculoskeletal demands associated with exercise activities [15,31], in order to appropriately “dose” the participants. In the present investigation, we calculated the JMOFs associated with the performance of 7 specific yoga poses in order to quantify their musculoskeletal demands in a sample of seniors, whose strength and flexibility capacities are undoubtedly less than those of average young-to-middle aged yoga practitioners. The JMOF profiles of these 7 asanas may ultimately be used to guide yoga instructors in the choice of poses that are well-balanced, target a variety of functionally important muscle groups and avoid overloading musculoskeletal structures.

Although net JMOFs have been used to quantify the musculoskeletal demands associated with a variety of exercise activities [32,33] this kinetic approach has inherent limitations. In calculating the JMOFs we used an inverse dynamics approach and thus do not account for co-activation of antagonistic muscle groups. Consequently, the actual internal (muscular) joint moments are likely to be underestimated. EMG analysis can be used to support the JMOF findings and in general the EMG results of the present study were in agreement with the kinetic data – muscle activities were low in those poses that had small JMOFs and high in poses generating higher JMOFs. In addition, poses with EMG activations lower than those generated during self-selected walking also generated JMOFs which were less than the average peak JMOFs produced during the walking trials.

When comparing the JMOFs generated during the yoga poses with the average peak JMOF generated during walking, it is important to consider that walking is a cyclic activity in which the JMOFs increase and decrease during a gait cycle. Thus, we calculated and recorded the *peak* JMOFs, across the hip, knee, and ankle, which were produced during the walking trials. In contrast, during both the yoga classes and the laboratory sessions, the participants held their poses static “for a full breath” before returning to a starting position, and we calculated the average JMOFs engendered during the middle 3-seconds of each pose. Thus, a fair comparison between the JMOFs engendered during yoga and walking should take into consideration the fact that peak JMOFs reported during walking only occur for an instant in time, whereas the average JMOFs produced during each yoga pose persist for more than 3 seconds. Consequently, although the peak JMOFs produced during dynamic activities such as walking may be greater than the average JMOFs generated during the yoga poses, the overall muscular stimulation (i.e. activation and loading) afforded during yoga posing may be greater than that produced during walking or other dynamic activities (e.g. resistance exercise). Additionally, it is important to note that because we limited our analysis to the static phase of the poses, we cannot directly extrapolate our findings to other vinyasa-based or “flowing” yoga styles that may use similar postures.

## Conclusions

We were able to quantify the lower extremity physical demands of 7 commonly practiced, minimally modified (from standard forms) standing yoga poses. This is a first step in the design of *evidenced*-*based* yoga programs (those in which poses are selected based on their known biomechanical profiles) intended accomplish one or more clinical goals. These goals may include targeting specific joints or muscle groups, addressing specific deficits in strength and muscular endurance, promoting improvements in physical function (e.g. balance), or unloading pathological tissues and structures at risk of injury. Goal-specific programs will need to be tested in randomized controlled-trial designs in order to determine whether they do accomplish the intended outcome(s). In addition to assessing the clinical effectiveness of evidence-based pose series, future studies should describe the physical demands of additional commonly-used poses, pose modifications, and the demand associated with between-pose transitions in order to expand our current knowledge base and provide additional options for the design of safe and effective yoga programs.

## Endnotes

^a^ Participants have provided their written consent for the use of the stills from the video for scientific and educational purposes.

## Abbreviations

YESS: Yoga Empowers Seniors Study; JMOF: Joint moments of force; EMG: Electromyography; GMED: Gluteus medius; HAMS: Hamstrings; VL: Vastus lateralis; GAS: Gastrocnemius.

## Competing interests

The authors declare that they have no competing interests.

## Authors’ contributions

MYW made substantial contributions to the study including study design, data acquisition, statistical analysis, results interpretation, and manuscript drafting. SY participated in data acquisition, data analysis, and result preparation. RH and SS helped on data acquisition, data analysis and manuscript revisions. LK designed and carried out the yoga program and participated in data collection. GA and GS significantly contributed to the study design, funding acquisition, study implementation, and critically revised the manuscript for important intellectual content. All authors read and approved the final manuscript.

## Study design

Comparisons of biomechanical demands among standing yoga poses: Descriptive/Comparative Laboratory Study.

## Pre-publication history

The pre-publication history for this paper can be accessed here:

http://www.biomedcentral.com/1472-6882/13/8/prepub
